# Supplementary material for: CB2 Receptor Stimulation and Dexamethasone Restore the Anti-Inflammatory and Immune-Regulatory Properties of Mesenchymal Stromal Cells of Children with Immune Thrombocytopenia
Source: Int J Mol Sci. 2019 Feb 28;20(5):1049. doi: 10.3390/ijms20051049 (PMC6429305; doi:10.3390/ijms20051049)
Supplement: Supplementary file 1 [file ijms-20-01049-s001.pdf]

CB2 Receptor Stimulation and Dexamethasone Restore the Anti-Inflammatory and Immune-Regulatory Properties of Mesenchymal Stromal Cells of Children with Immune Thrombocytopenia

Supplementary Materials

Table S1. Annexin-V and PI double-stained Apoptosis Assay, in MSCs derived from 2 ITP patients after 24h treatments with JWH-133 [2,5 μM] and AM630 [1 μM], alone and in combination. The table shows the percentage of total apoptotic cells. A Wilcoxon test has been used for statistical analysis. $p \leq 0.05$ has been considered statistically significant. * vs NT (NT).

	Percentage of total apoptotic ITP-MSCs			
	Treatments			
Samples	NT	JWH-133	AM630	J+A
MSC ITP-1	45,88	30,59*	52,01*	55,36*
MSC ITP-2	46,22	31,70*	53,60*	55,90*

Table S2. (A) Viability of T cells co-cultured with ITP-MSCs estimated by a cytofluorimetric assay after 24h treatment with JWH-133 [2,5 μM] and AM630 [1 μM], alone and in combination. The table shows the results, as cell number per 10^6. A Wilcoxon test has been used for statistical analysis. $p \leq 0.05$ has been considered statistically significant. * vs T cells+MSC (B) TNF-α release by ITP-MSCs and T cells investigated by ELISA assay after 24h treatment JWH-133 [2,5 μM] and AM630 [1 μM], alone or in combination. The table shows the concentrations [pg/mL] of TNF-α. A Wilcoxon test has been used for statistical analysis. $p \leq 0.05$ has been considered statistically significant. * vs MSC.

A)

T Cell Viability					
Sample 1	T cells	T cells+MSC	T cells+MSC JWH-133	T cells+MSC AM630	T cells+MSC J+A
(n° cells X 10^6)	5,86	6,26	4,36*	6,61	6,62
Sample 2	T cells	T cells+MSC	T cells+MSC JWH-133	T cells+MSC AM630	T cells+MSC J+A
(n° cells X 10^6)	5,74	6,18	4,43*	6,58	6,38

B)

TNF-α				
Sample 1	MSC-ITP NT	MSC+LPS	T cells	T cells+LPS
pg/mL	0,51	0,99*	5,88*	7,62*
Sample 2	T cells	T cells+MSC	T cells+LPS	T cells+MSC LPS
pg/mL	0,39	1,08*	5,67*	7,86*
